# Is treatment with interferon-*α* effective in all patients with metastatic renal carcinoma? A new approach to the investigation of interactions

**DOI:** 10.1038/sj.bjc.6601622

**Published:** 2004-02-17

**Authors:** P Royston, W Sauerbrei, A Ritchie

**Affiliations:** 1 Cancer Division, MRC Clinical Trials Unit, 222 Euston Road, London NW1 2DA, UK; 2 IMBI, University Hospital of Freiburg, Freiburg, Germany; 3 Urology Department, Gloucestershire Royal Infirmary, Great Western Road, Gloucester GL1 3NN, UK

**Keywords:** metastatic renal carcinoma, subgroup analysis, interactions, fractional polynomials, interferon

## Abstract

The first analysis of the MRC RE01 trial in metastatic renal carcinoma identified a 28% reduction in the hazard of death for patients treated with interferon-*α* compared with medroxyprogesterone acetate (MPA). No subgroup was identified in which treatment with interferon-*α* was more or less effective than MPA. We used a new approach based on fractional polynomials to investigate the updated data from this trial for the possible interaction of treatment with prognostic factors. In the spirit of hypothesis generation, we considered 10 possible prognostic variables, of which white cell count (WCC) was found to influence the effectiveness of interferon treatment. In patients treated with MPA, there was no prognostic effect of WCC, whereas, in patients treated with interferon, the risk of dying increased significantly with WCC level. We defined subgroups of patients based on WCC levels and estimated a hazard ratio of 0.53 in favour of interferon in patients with WCC <6.5 × 10^9^, whereas for patients with WCC >10 × 10^9^ the risk appears to be similar between the treatment groups, or even slightly raised in the interferon group. Since our results are derived from flexible statistical models, they may be interpreted as a new hypothesis and require validation in independent data.

Between 1992 and 1997, 350 patients with metastatic renal carcinoma were randomised to enter the MRC RE01 trial comparing interferon-*α* with medroxyprogesterone acetate (MPA) at 31 centres in the UK. In the first paper based on 335 patients and 236 deaths, a 28% reduction in the risk of death in the interferon-*α* group was reported (MRCRCC, 1999). The trial was stopped early, because the treatment effect crossed the effectiveness boundary in a triangular sequential design. Owing to the side effects of the interferon-*α* regimen, it is important to investigate whether the clear overall survival advantage is present in all patients. In the initial report (MRCRCC, 1999), there was no evidence from *χ*^2^ tests of heterogeneity that any prognostic factor had an influence on the effectiveness of interferon-*α*. Thus, no subgroup was identified in which the treatment was more or less effective.

Updated data with 322 deaths were analysed by using a new approach to modelling interactions between treatment and continuous covariates (Royston and Sauerbrei, 2003). We systematically investigated whether any of the potential prognostic factors recorded at randomisation exhibits any predictive value, meaning that the effect of treatment depends on such a factor.

Details of results on further end points such as progression-free survival may be found in the first paper (MRCRCC, 1999). Here we will consider only the primary end point of the trial, overall survival. In this investigation, we considered age, WHO performance status and other clinical and laboratory features listed in Table 2. With the exception of weight loss, erythrocyte sedimentation rate (ESR) and nuclear grade, this list includes the ‘standard’ prognostic factors for advanced renal cell carcinoma (Elson, 2003).

The data were reanalysed, not to test a specific interaction between treatment and a factor (if significant, often called a predictive factor), but more with an emphasis on data exploration. We used a statistical method based on fractional polynomials for the investigation of potential predictive factors. It extends the multivariable fractional polynomial (MFP) method used to investigate the simultaneous prognostic effect of several continuous factors. The main advantages of analysis of continuous factors with fractional polynomials are to extract more information from such factors, to improve the statistical power to detect influential variables and their interaction with treatment (Farewell *et al*, 2003), and to circumvent the problems of arbitrary categorisation (Altman *et al*, 1994; Royston and Altman, 1994; Sauerbrei and Royston, 1999; Royston and Sauerbrei, 2003).

Clearly, the results of this unplanned analysis may only be interpreted in a hypothesis-generating sense and the findings require validation in independent data (Assmann *et al*, 2000).

## PATIENTS AND METHODS

### Patient characteristics and treatment

The principal eligibility criterion was a histologically or cytologically proven metastatic renal carcinoma in patients of WHO performance status 0, 1 or 2 with at least one measurable metastatic lesion. Patients were assigned at random to interferon-*α* (Intron-A, Schering-Plough) 10 MU, by subcutaneous injection three times per week for 12 weeks, or MPA (Provera, Upjohn) 300 mg by mouth each day for 12 weeks. The present analysis is based on data updated to June 2001.

Overall survival (OS) was calculated from the date of randomisation to the date of death, irrespective of the cause of death. The median follow-up time is 56 months. Of 350 patients randomised, no follow-up information is available for three patients. By June 2001, 322 (93%) patients were known to have died.

Reasonably complete baseline data were available for the following 14 potential prognostic factors: age, sex, nephrectomy status, WHO performance status, time from initial diagnosis of renal carcinoma to randomisation, time from diagnosis of metastasis to randomisation, single/multiple sites of metastasis, haemoglobin, white cell count, serum calcium, ESR, viscosity, histopathological tumour grade and body weight.

For further details of patient characteristics, treatment and follow-up, see MRCRCC (1999).

### Statistical analysis

Before starting the analysis, we decided to include in the investigation all potential prognostic factors for which the proportion of missing values was less than 15%, yielding 10 variables. To maximise the statistical power by including all patients in the multivariable analysis, we used the method of van Buuren *et al* (1999) to impute the missing values. As a sensitivity analysis, we repeated the multivariable analysis for the subset of patients with complete data for all prognostic variables.

The potential effects of prognostic factors were investigated in univariate analyses. Continuous factors were modelled by using fractional polynomials (Royston and Altman, 1994; Sauerbrei *et al*, 1999). All survival analyses and tests of hypotheses were conducted within the Cox regression framework. *P*-values were based on the partial likelihood ratio test. The proportional hazards assumption was assessed by the Grambsch–Therneau test (Grambsch and Therneau, 1994). The only variable showing violation of proportional hazards was WHO performance status; however, this had no major impact on the remaining analyses and is not considered further.

A simultaneous assessment of the effects of prognostic factors and of the predictive value of these factors (i.e. of their interaction with treatment) was performed within a multiple regression framework. We used the MFPI algorithm (an extension of the MFP algorithm, see Appendix A) with a nominal *P*-value 0.05, to determine the prognostic factors with influence on OS and to investigate whether any of the 10 factors had a significant interaction with treatment (i.e. are predictive factors).

As a check of any interactions identified by this method and for the purpose of presentation, we divided the patients into four groups, according to a categorisation of each predictive factor. In the subgroups, comparison of the Kaplan–Meier curves for the treatments and the corresponding estimates of the treatment effect should broadly agree with the continuous function representing the effect of treatment from the MFPI analysis. The primary cut-point was chosen in a data-dependent fashion, according to the function estimated by the MFPI analysis. Two other cut-points were chosen among the quartiles of the distribution of the predictive factor in question. This gives three cut-points and four groups for each continuous variable. Each analysis of interactions was adjusted for other prognostic factors determined at the initial step by the MFPI algorithm (see Appendix A).

## RESULTS

The patients' ages lay between 45 and 72 years (median 60). About two-thirds were male. WHO performance status was one in about 50% of the patients and 0 and 2 in about 25% each. The median time from first metastases to the date of randomisation was 1.2 months, and median time from primary diagnosis to randomisation was 3.2 months. In all, 57% had had a nephrectomy and 84% of patients already had multiple metastases on entering the trial. Further information on these variables including treatment details may be found in the first report of the study. Table 1

**Table 1:** Baseline characteristics of blood parameters

**Characteristic**	**MPA (*n*=175)**	**Interferon-*α* (*n*=172)**
*Haemoglobin (g dl*^*−1*^)		
<11	44 (25%)	46 (27%)
11–12.5	36 (21%)	52 (30%)
12.5–14	48 (27%)	31 (18%)
>14	32 (18%)	35 (20%)
Unknown	15 (9%)	8 (5%)
		
*White cell count (× 10*^*9*^* l*^*−1*^)		
<6.5	34 (19%)	47 (27%)
6.5–8	43 (25%)	41 (24%)
8–10	39 (22%)	40 (23%)
>10	44 (25%)	36 (21%)
Unknown	15 (9%)	8 (5%)
		
*Serum calcium (mmol l*^*−1*^)		
<2.3	24 (14%)	40 (23%)
2.3–2.4	32 (18%)	27 (16%)
2.4–2.5	43 (25%)	49 (28%)
>2.5	49 (28%)	42 (24%)
Unknown	27 (15%)	14 (8%)

gives details of three variables not considered in the first report. All factors are well balanced between the treatment groups. Missing values were replaced with imputations to give complete data for analysis of results from all 347 patients.

Table 2

**Table 2:** Univariate analysis of prognostic factors and treatment (Cox regression)

**Characteristic**	***β***	**s.e.**	***P*-value**
Age^a^	0.0026	0.0053	0.6

*Sex*			
Male	0	—	0.8
Female	−0.04	0.12	
			
*WHO performance status*			
0	0	—	<0.001^b^
1	0.32	0.14	
2	1.12	0.16	
			
Months since first diagnosis of renal carcinoma^a^	−0.0040	0.0016	0.02
Transformed months since first diagnosis of metastases^c^	−0.19	0.04	<0.001
			
*Nephrectomy*			
No	0	—	0.07
Yes	−0.21	0.11	
			
*Metastases*			
Single	0	—	0.3
Multiple	0.17	0.15	
			
Transformed haemoglobin^d^	35.9	4.4	<0.001
White cell count^a^	0.087	0.013	<0.001
Serum calcium^a^	0.99	0.29	0.001
			
*Treatment*			
MPA	0	—	0.009
Interferon-*α*	−0.29	0.11	

**Figure 1:**
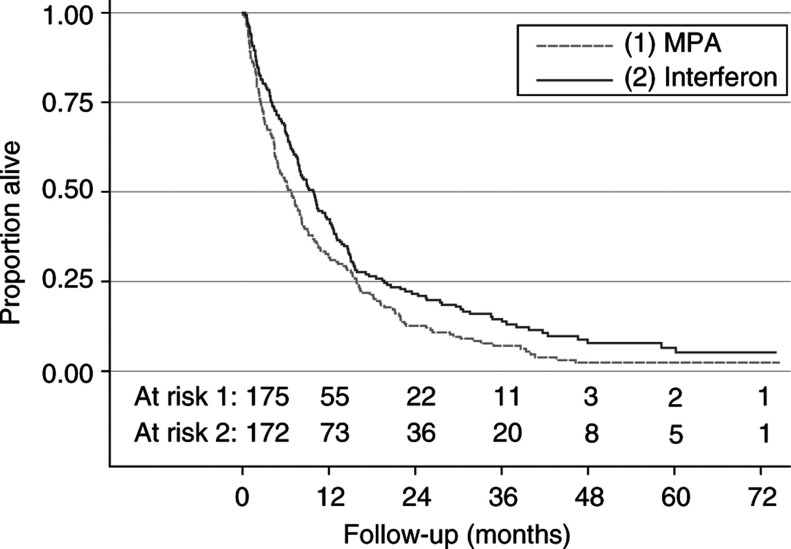
Kaplan–Meier estimates of overall survival by treatment group.

Values of *β* are log hazard ratios. s.e.=standard error.

a Linear function.

b Trend test.

c Log transformation.

d 1/haemoglobin.

shows the results of a univariate investigation of the prognostic factors and of treatment. The estimated effect of treatment changed little from the earlier analysis (MRCRCC, 1999). The hazard ratio for the risk of dying, favouring interferon-*α*, was 0.75 (CI 0.60–0.93) compared with 0.72 before. The effect of treatment is shown by Kaplan–Meier survival curves in Figure 1

. Of the prognostic factors investigated, WHO performance status, haemoglobin, white cell count, time since metastases and time since primary diagnosis showed a significant effect on the survival time. Fractional polynomial analysis yielded significantly nonlinear functions for time from metastases to randomisation (log transformation) and haemoglobin (reciprocal transformation).

Multivariable assessment of prognosis with deletion of nonsignificant prognostic factors resulted in a model comprising WHO performance status, haemoglobin, white cell count and time from metastases to randomisation. All these variables were highly significant in univariate analysis. The months since diagnosis and serum calcium were significant in the univariate analysis, but no longer so in the multivariable model (*P*>0.5). After adjustment for other factors, only the effect of time from metastases to randomisation required a nonlinear function. The function (not shown) suggests an elevated risk of death only for patients with intervals from diagnosis of metastases of <3 months. A sensitivity analysis using only the 306 patients with complete data on all prognostic factors gave the same multivariable model.

**Figure 2:**
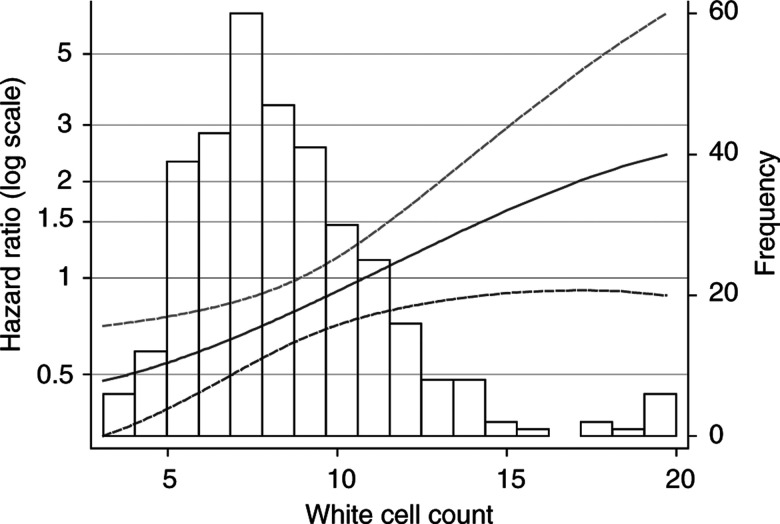
Dependence on white cell count (× 10^9^ l^−1^) of the effect of treatment with interferon-*α* compared with MPA, estimated from fractional polynomial model. Solid line: hazard ratio; dashed lines: 95% CI for hazard ratio. The horizontal line at hazard ratio=1 denotes equivalence of treatment effects. Values beneath this line indicate that interferon-*α* is more effective than MPA, and *vice versa*. A bar chart showing the distribution of white cell count values for all patients in the study is superimposed.

Adjusting for factors in the multivariable model, we investigated all the 10 prognostic variables given in Table 2 for a possible interaction with treatment. A highly significant interaction was found with white cell count (*P*=0.0001, MFPI procedure). No further interactions were significant at the 5% level. Appendix B gives the mathematical details of the interaction model. A significant prognostic effect of WCC is seen only in the patients treated with interferon. How the treatment effect of interferon-*α* compared with MPA, estimated from our model, changes with the patient's white cell count is of considerable clinical relevance (see Figure 2

). For patients with very low white cell counts, the risk of dying appears to be substantially reduced by interferon-*α*, whereas, for those with values around 10, the risk seems to be similar for the two treatments. For the 25% of patients with white cell count larger than 10, interferon-*α* seems to be of no benefit, and the risk may even be somewhat raised.

**Figure 3:**
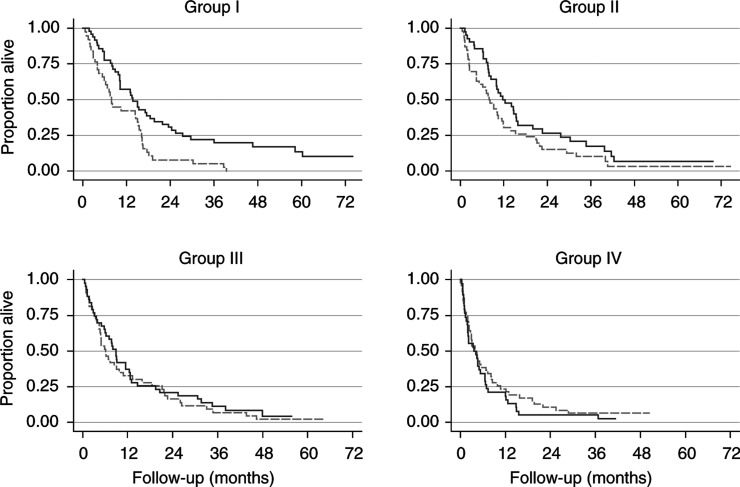
Survival by subgroups I–IV of white cell count (× 10^9^ l^−1^). Definition of groups according to white cell count: I: <6.5; II: 6.5–8; III: 8 – 10; IV: >10. Solid lines: interferon-*α* group; dashed lines: MPA group.

As a visual check of the results from our complex modelling procedure, we divided the patients into four groups according to white cell count, by using cut-points of 6.5, 8 and 10 × 10^9^ l^−1^. The value of 10 was chosen because the beneficial effect of treatment disappears at about this point (Figure 2). The other two cut-points represent the first two quartiles of the distribution of white cell count. Figure 3

shows the estimated survival curves by treatment in the four subgroups of white cell count.

In accordance with the function shown in Figure 2, a trend is seen towards a substantial survival advantage of interferon-*α* in group I with the lowest white cell counts, becoming weaker in subgroups II and III with larger counts, and no longer present in group IV. The estimated hazard ratios for the effect of interferon-*α* in comparison to MPA (with 95% CI) are 0.53 (0.34–0.83), 0.69 (0.44–1.07), 0.89 (0.57–1.37) and 1.32 (0.85–2.05) in white cell count groups I–IV, respectively. These estimates are little influenced by adjustment for WHO performance status, haemoglobin and time since metastases (details not given). In a sensitivity analysis using only the 306 patients with complete data on all prognostic factors, the principal results described above were confirmed.

## DISCUSSION

The first report of the MRC RE01 study (MRCRCC, 1999) showed that interferon-*α* prolongs survival, but no factors predictive of response to treatment with interferon-*α* were identified. Owing to the toxicity profile of interferon-*α* and its acceptance as standard therapy for metastatic RCC in the UK, it is important to investigate whether all patients in fact benefit from this therapy. With the data updated to June 2001, we confirmed the overall treatment effect. More importantly, we were able to identify an interaction between treatment and white cell count. The statistical power of this new analysis is improved, since the number of events (deaths) is 36% greater than before. Furthermore, we used a new approach to investigate interactions. The usual approach of categorising continuous variables reduces the power, and, more importantly, the results depend on the choice of a ‘suitable’ cut-point. By contrast, the MFPI approach estimates functions to describe the influence of the variable in question on the outcome in each treatment group, and does not require the selection of any cut-points.

As in the first report, we investigated the prognostic and predictive value of sex, age, performance status, time since diagnosis of renal carcinoma, time since diagnosis of metastases, nephrectomy status and number of metastatic sites (single/multiple). In addition, the prognostic and predictive value of haemoglobin, white cell count and serum calcium were investigated. For these measurements, we handled the issue of data missing in some patients by using an imputation approach (van Buuren *et al*, 1999). By multivariable modelling with the MFP approach, we identified haemoglobin and white cell count as strong prognostic factors in addition to WHO performance status and time from metastases to randomisation. With the MFPI approach, white cell count was identified also as a predictive factor. The level of white cell count appears to influence the effectiveness of treatment with interferon-*α* (Figures 2 and 3). Patients with low white cell counts seem to have a reduced risk of dying following interferon-*α* treatment, but the risk increases with the count. By contrast, the estimated risk function for MPA changes little with a change in white cell count.

An understanding of the relationship between the pretreatment white cell count and the risk of dying following interferon treatment is hampered by limited knowledge of the mechanism of action of interferon. A number of possible anticancer mechanisms have been proposed, including direct antiproliferative effects, modulation of class I and II MHC antigen expression, enhanced natural killer (NK) cell activity, modulation of haematopoiesis and inhibition of angiogenesis (Small and Motzer, 2003).

Negative regulation of host cytotoxicity has been reported to be mediated by certain prostaglandins and a variety of cell types including macrophages and neutrophils (Kimber and Moore, 1985). It is possible that some patients in our study had raised white cell counts as a result of raised neutrophil counts, and that these cells are responsible for blocking cytotoxic enhancement by the interferon. Lymphopaenia has been shown to correlate with advanced disease stage in Hodgkin's disease. It is not clear if the lymphopaenia is due to lymphocyte trafficking to sites of disease or to an absolute quantitative deficiency (Ayoub *et al*, 1999). Unfortunately, our database recorded only the total white cell count; we did not have data on the constituent neutrophil and lymphocyte counts. Our hypothesis that adverse outcome is associated with raised neutrophil counts will be tested by more detailed analyses of the peripheral blood white cells in future interferon studies. Longitudinal studies, of the effect of interferon therapy on white cell subpopulations in peripheral blood and within tumour tissue, may also provide clues to the mechanism of action.

In previous studies, various prognostic factors have been identified as important indicators for survival of patients with metastatic renal cell carcinoma. These include performance status, recent weight loss, disease-free interval, pretreatment ESR, lactate dehydrogenase (LDH), neutrophils, haemoglobin, extrapulmonary and bone metastases, and the number of metastatic sites (Elson *et al*, 1988; Palmer *et al*, 1992; Lopez-Hänninen *et al*, 1996; Gelb, 1997; Culine *et al*, 1998; Hoffmann *et al*, 1999; Motzer *et al*, 1999, 2002). A recent study of 425 good performance status patients added C-reactive protein (CRP) to this list and presented a model based on neutrophils, CRP, LDH, time from primary diagnosis to metastases, number of metastatic sites and presence of bone metastases (Atzpodien *et al*, 2003). Negrier *et al* (2002) analysed the details of 782 patients enrolled in successive multicentre trials using cytokine regimens. They found that biological signs of inflammation, an interval of less than 1 year from diagnosis to metastases, elevated neutrophil counts, liver metastases, bone metastases, performance status, the number of metastatic sites, alkaline phosphatase and haemoglobin levels were predictive of survival outcome. Elevated neutrophil counts were also identified as one of four predictors of rapid progression of the disease. However, as with many diseases, a great many prognostic factors are proposed, but most await confirmation of their importance in the context of a multivariable model.

As with all complex and flexible statistical modelling procedures, the MFPI approach may sometimes produce results which are strongly influenced by pecularities in the data, for example, outliers or influential points (Belsley *et al*, 1980). Single patients or a small group of patients may determine the shape of the function or seriously influence the *P*-value of a test statistic. Therefore, a check of the main results and a sensitivity analysis of the major assumptions are required. In our analysis of interactions, we found that two extreme values of white cell count (37.1 and 55.2 × 10^9^ l^−1^) determined the shape of the function in a region of no interest (white cell count >25, function not shown) and influenced the *P*-value for interaction. On deleting these extreme cases, the *P*-value changed from 0.0001 to 0.008, still strong evidence of an interaction between treatment and white cell count. The more essential check of the functions proposed is to verify the result from MFPI, by considering treatment comparisons within subgroups derived by inspecting the MFPI functions. The subgroups have an order allowing a sensible check of the continuous function postulated for the treatment effect. A comparison of the treatment effects across four subgroups defined by white cell count clearly demonstrates that the interaction postulated by our model is not a statistical artefact.

Despite the checks considered in the paper, the interaction between treatment and white cell count can only be interpreted in a hypothesis-generating sense (Assmann *et al*, 2000). This analysis was not planned in advance, and we investigated as many as 10 factors for possible interaction with treatment. The interaction found in our data may conceivably be due to chance, and needs validation in independent studies before it can confidently be used to guide treatment decisions. If our finding is confirmed, the usefulness of treatment with interferon-*α* has to be questioned for patients with higher white cell counts. Confirmation of the prognostic role of white cell count in patients treated with interferon-*α* may be obtained when results are available from the ongoing RE04 trial of interferon monotherapy *vs* the Atzpodien triple regimen (interferon-*α*, interleukin-2 and 5-fluorouracil) in patients with good WHO performance status.

The potential outcome of the findings of this study is that a simple, widely available laboratory test may be able to select patients likely to benefit from interferon therapy. Clearly, much further investigation of the mechanism of action of interferon is required 20 years after its initial use in renal cancer (Quesada *et al*, 1983).

## Appendix A: The MFPI algorithm

Royston and Sauerbrei (2003) proposed a method of analysing treatment/prognostic factor interactions with data from clinical trials. The analysis of interactions with categorical variables such as sex and performance status is well established and reasonably straightforward. Typically, in survival data, the hypothesis test of no interaction is assessed within a Cox model or by a stratified logrank test, and the hazard ratio for the treatment effect and its CI is estimated at each level of the prognostic factor. In a situation with no predefined hypothesis, *P*-value adjustment for multiple testing may be made to counteract the inflated probability of a type I error.

How to approach such an analysis for a continuous prognostic factor is not so simple. The conventional method has been to categorise the factor into two or more groups and apply the analysis just described, perhaps with a suitable test for trend. However, this method may lack power (Farewell *et al*, 2003) and the results will depend on the choice of cut-point(s). Alternatively, linearity may be assumed and the slopes may be compared, but power will again be lost if the relationship is not in fact linear.

The MFPI algorithm is based on fractional polynomial analysis of continuous predictors (Royston and Altman, 1994; Sauerbrei *et al*, 1999) and is an extension of the MFP algorithm of Sauerbrei and Royston (1999). In the MFP algorithm, continuous predictors are subject to possible transformations including logarithm, square root, etc, or to two such transformations, giving a fractional polynomial model of degree 2 (FP2) (see Sauerbrei *et al* (1999) for an example in breast cancer explaining fractional polynomial analysis in detail). Transformation(s) are applied only if the fit of the model is significantly improved, the default being a linear model. Each predictor is examined in turn and the process stops when no change in the required transformation takes place. At the same time, a predictor may be eliminated from the model if it is not statistically significant.

The MFPI algorithm proceeds in two stages. First, a multivariable adjustment model is developed from all the available prognostic factors by using the MFP algorithm as just outlined. Then, each candidate continuous factor is modelled by an FP2 function in each treatment group, allowing for possible confounding by including the variables of the adjustment model. This approach allows the functional influence of the factor to differ by treatment group, and is equivalent to saying that the treatment effect varies continuously with the level of the prognostic factor.

For the purposes of presentation, two graphs may be given. The first graph shows the estimated function in each treatment group. The second (the more important) shows the difference between the estimated functions, which, as just mentioned, is a representation of the continuously varying treatment effect. These graphs will often suggest natural cut-point(s) on the factor, which should be confirmed by Kaplan–Meier plots of survival probabilities according to the groups defined by the cut-point(s).

## Appendix B: Further details of the interaction model

The MFPI procedure indicated a fractional polynomial transformation with powers 2 and 3 for white cell count (WCC, *x*), of the form *β*_1_*x*^2^+*β*_2_*x*^3^. The equations for log hazard ratio (log HR) derived from the interaction model are as follows (standard errors are in parentheses). In the MPA treatment group:

In the interferon-treated group:

The division of WCC by 10 in the above formulas makes them more readable, but has no effect on the functions estimated for WCC. As may be judged by comparing the regression coefficients for (WCC/10)^2^ and (WCC/10)^3^ with their respective standard errors, a significant prognostic effect of WCC is seen only in the patients treated with interferon.
